# A prospective risk analysis for the clinical commissioning of a dose-driven continuous scanning proton therapy system

**DOI:** 10.3389/fonc.2026.1826522

**Published:** 2026-05-20

**Authors:** Steven Herchko, Xiaoying Liang, Jiajian Shen, Chris Beltran, Keith M. Furutani

**Affiliations:** 1 Department of Radiation Oncology, Mayo Clinic Florida, Jacksonville, FL, United States; 2 Department of Radiation Oncology, Mayo Clinic Arizona, Phoenix, AZ, United States

**Keywords:** clinical commissioning, dose-driven continuous scanning, failure mode and effects analysis, process mapping, proton beam therapy

## Abstract

**Introduction:**

Proton dose driven continuous scanning (DDCS) is a form of proton pencil beam scanning (PBS) in which the beam may remain on during transitions between successive spots. Mayo Clinic Florida (MCF) is preparing to commission a synchrotron-based proton therapy system designed for DDCS, incorporating novel irradiation control features. Given the novelty and complexity of this system, a systematic risk-based analysis was performed to inform upcoming commissioning strategies.

**Methods:**

A prospective risk analysis was conducted using the American Association of Physicists in Medicine (AAPM) Task Group 100 (TG 100) framework. A comprehensive process map of the DDCS irradiation control workflow was developed. Failure modes and effects analysis (FMEA) was performed to identify potential delivery system failure modes and to score each failure mode based on occurrence, severity, and lack of detectability. Targeted mitigation and commissioning strategies were then developed to address the highest-risk items.

**Results:**

Twenty-nine delivery system failure modes were identified. The highest-risk failure modes were specific to DDCS operation. Targeted commissioning strategies were defined, including scan path measurement and modeling, validation of move and flap dose, dose monitor collection efficiency testing, spot position monitor performance evaluation, and verification of control system intervention behavior. Incorporation of these strategies into the commissioning plan reduced the mean RPN by more than 50%.

**Discussion:**

This work provides a detailed description of the MCF proton DDCS irradiation control system and uses a TG 100 based approach for identifying system-specific risks. As new systems incorporate technologies for which limited guidance exists, institutions must perform their own risk analyses to determine appropriate quality management strategies.

## 1 Introduction

Proton beam therapy is a valuable treatment technique that can provide both dosimetric benefits and improved clinical outcomes when compared to conventional photon beam therapy (1–3). Pencil beam scanning (PBS) is currently the most used form of proton beam therapy in which a pencil beam of protons is delivered to planned nodes or “spots” within the patient. The dose contribution from the pencil beam at each spot is summed to determine the overall dose distribution within the patient.

Proton PBS can be delivered using discrete spot scanning or dose-driven continuous scanning. Discrete spot scanning (DSS) only delivers dose at the designated spot positions, and no dose is delivered as the beam moves from one spot position to another. DSS has been widely implemented and is the most common form of proton PBS delivery. Dose-driven continuous scanning (DDCS), also known as raster scanning, is an alternative PBS delivery technique in which the beam is allowed to remain on between successive spots. DDCS delivers dose both at the spot position and between spots as the scanning magnets move the beam from one spot to the next. Compared to DSS, DDCS may allow for a more efficient beam delivery since the beam can remain on and the time required to turn the beam on and off between successive spots can be eliminated (4, 5). DDCS also presents unique challenges as the temporal characteristics of beam delivery related to the beam scanning path and beam current fluctuations influence the delivered dose distribution (6–11).

Mayo Clinic Florida (MCF) is in the process of installing a new synchrotron-based proton therapy system designed for proton DDCS delivery. While DDCS has been widely utilized in Carbon ion therapy, its use in proton therapy has been limited (12–17). In addition to DDCS delivery, the system will include other unique beam features such as a high dose rate, small minimum spot MU, small minimum spot period, and small spot size. Additionally, unique irradiation control system features such as a skip spot, on fly break spot, and skipped spot position monitor (SPM) judgment will be implemented in our system for the first time to improve delivery efficiency by reducing beam delivery disruptions or system aborts (18). A list of DDCS-specific and MCF-specific definitions that may not be familiar to all readers is provided in section 2.1, and details of these unique features are listed in **Tables 1** and **2** and discussed in the methods section.

**Table 1:** Key unique beam and irradiation control features of the upcoming Mayo Clinic Florida (MCF) synchrotron-based proton therapy system compared with the existing proton therapy system at Mayo Clinic Arizona (MCA) from the same vendor.

Feature	MCF	MCA
Delivery approach	Dose-driven continuous scanning (DDCS)	Discrete spot scanning (DSS)
High dose rate	up to 40 MU/s*	8 MU/s
Small minimum spot MU	0.1 mMU	3 mMU
Small minimum spot period	<30 µs	1100 µs
Small spot size	~2 mm @ 230 MeV	~2 mm @ 230 MeV

*1 MU corresponds to approximately 0.4 - 0.9×10^9^ protons, depending on beam energy (70–230 MeV) (28).

**Table 2:** Key unique beam delivery scenarios accounted for in the upcoming Mayo Clinic Florida synchrotron-based proton therapy system compared with other DDCS delivery systems.

Scenario	MCF	Other
Spot MU attained during the move between spots	Skip Spot/On fly break spot	Abort
Stop MU < Qmon	Skip SPM judgement, continue beam delivery	Abort
Cumulative Skipped SPM Judge MU > Threshold 1 (0.283 MU)	On fly break spot	N/A
Cumulative Skipped SPM Judge MU > Threshold 2 (0.291 MU)	Abort	N/A

These new features pose challenges in the treatment delivery irradiation control system. In preparation for the commissioning and clinical implementation of this new system, a systematic risk analysis was performed to identify and mitigate potential risks in the proposed MCF proton DDCS treatment delivery workflow, specifically focusing on the irradiation control system, utilizing the methodology of the American Association of Physicists in Medicine (AAPM) Task Group 100 (19). TG 100 proposes a prospective risk analysis approach that incorporates process mapping and failure modes and effects analysis (FMEA) to clearly define a process and then find potential risks (or failure modes) in the process. The impact of each failure is then quantified based on the occurrence (O), severity (S), and lack of detectability (D) of the failure mode. A risk priority number (RPN) for each failure mode is calculated and serves as the relative risk of each failure mode to allow for prioritization of intervention efforts to focus on the highest risk steps in the process. FMEA has been increasingly recognized as an effective approach for systematically identifying, prioritizing, and mitigating risks in complex clinical processes. In proton therapy, several studies have applied FMEA to treatment planning and clinical workflows (20–23). However, to the best of our knowledge, no published work has specifically addressed its application to the treatment delivery control system.

The prospective nature of this risk analysis approach lends itself particularly well to proton therapy systems such as ours, which incorporate novel beam and irradiation control system features implemented for the first time and for which no professional organization recommendations currently exist for commissioning, clinical implementation, and ongoing quality assurance. As new and emerging technologies and techniques, such as proton DDCS, outpace the dissemination of guidance documents, each institution must perform their own risk analysis based on the clinical workflow of their institution and technical specifications of their system to determine the appropriate quality management program.

## 2 Methods

### 2.1 MCF DDCS irradiation control system overview and definitions

**Figure 1:**
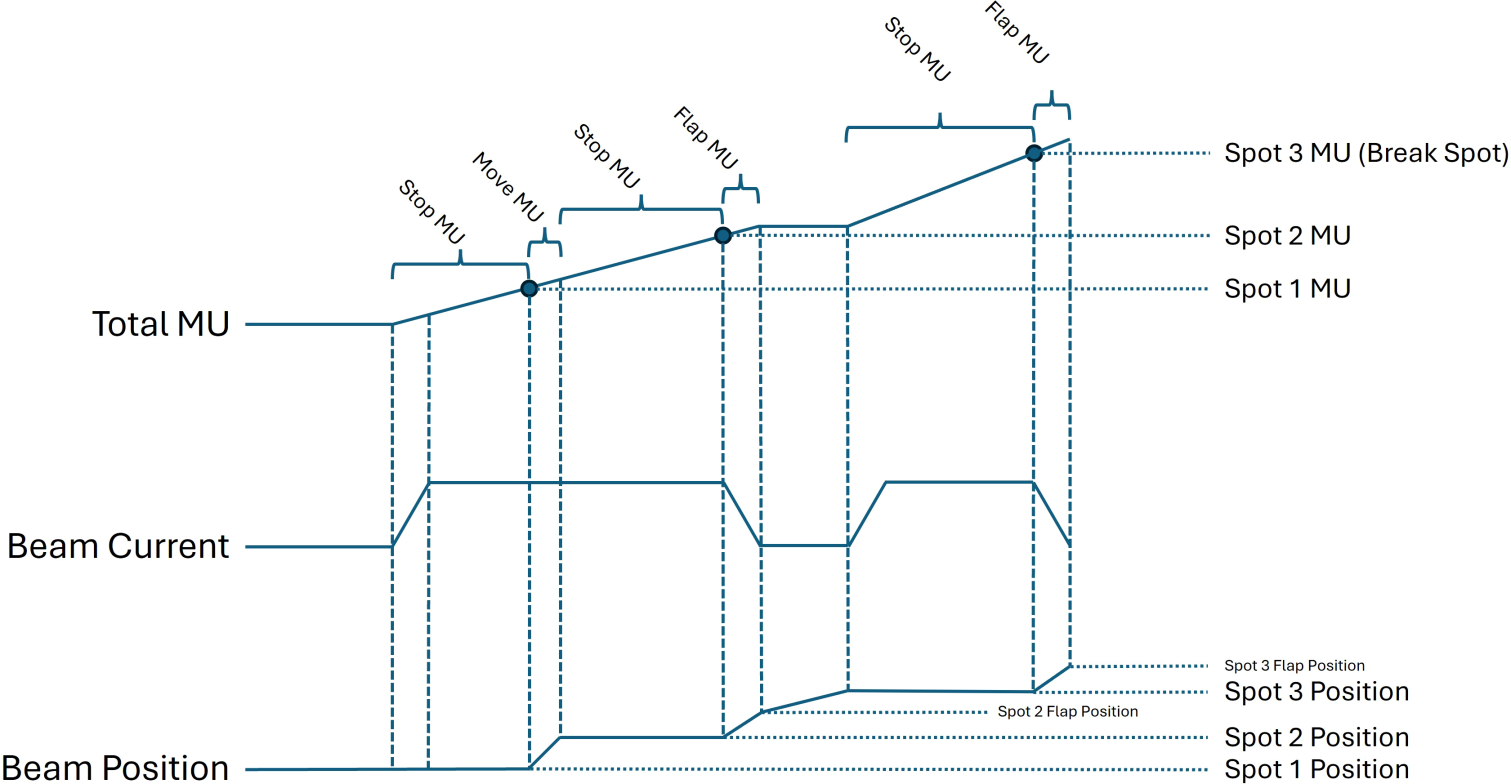
Relationship of total MU, beam current, and beam position for MCF proton DDCS treatment delivery for an example layer consisting of three spots. Note that magnitudes are not to scale and are for illustrative purposes only.

**Figure 1** depicts an example of MCF proton DDCS delivery for a layer consisting of three spots. The delivery of the first spot is identical to a DSS approach, in which the beam is not turned on until the planned spot position is reached. This results in the entirety of the spot MU being delivered at the planned spot position. For the subsequent continuously delivered spot (spot 2), the spot MU is delivered both while the beam is moving between spots (Move MU) and while the beam is at the planned spot position (Stop MU).

In the example shown, spot 3 is a break spot. This means that the beam is turned off after completion of MU delivery for spot 2. In DDCS delivery, break spots are introduced to control the transit dose between spots. As the beam ramps down before a break spot, a small amount of additional MU is delivered near the position of spot 2 (Flap MU) and the scanning magnet currents are adjusted to position the beam for the next spot. Once the planned position of spot 3 is reached, the beam is turned on and the beam current ramps back up to resume treatment delivery. After the delivery of spot 3 MU, the beam is again turned off, and a small amount of additional MU is delivered near the last spot position in addition to the planned spot MU.

The beam delivery dose monitoring system includes two types of detectors: two dose monitors (DM) that measure delivered MU, and a spot position monitor (SPM) that measures the position and full width at half maximum (FWHM) for each spot. The irradiation control system relies on real-time feedback from the dose monitoring system to control beam delivery and to ensure that the prescribed MU are delivered accurately and safely.

SPM judgements may be skipped if sufficient MU or time are not measured by the SPM to provide an accurate measurement for an SPM judgement. Successive spots may be skipped if the cumulative MU delivered since the last SPM judgement does not reach a pre-defined threshold. The cumulative skipped SPM judge MU thresholds (0.283 MU and 0.291 MU) were derived from the system requirement to limit the maximum dose delivered without valid monitoring, consistent with IEC safety principles for radiotherapy beam delivery. The upper threshold (0.291 MU) corresponds to the MU equivalent of 0.25 Gy under the system calibration and defines the Abort limit. The lower threshold (0.283 MU) is set to trigger a pre-emptive on-the-fly interruption (“on fly break spot”) with an added safety margin. This margin accounts for control and detection latency, ensuring that the total unmonitored delivered dose cannot reach or exceed the 0.291 MU limit. Together, these thresholds establish a layered control strategy that both guarantees compliance with the maximum allowable unmonitored dose and minimizes unnecessary aborts.

To facilitate a clear understanding of our irradiation control system, a list of DDCS and/or MCF-specific system specific terms that may not be familiar to all readers is provided below.

- Move MU – Monitor units (MU) delivered during DDCS delivery while the beam is moving from one spot to the next spot, Move MU are not delivered at the planned spot position.
- Stop MU – MU delivered during DDCS delivery while the beam is stationary at the planned spot position.
- Spot MU – planned MU for a given spot, Spot MU is the sum of the Move MU and Stop MU.
- Flap MU – MU delivered while beam current ramps down after the control system executed beam off. In our control system, beam delivery is terminated using a steering magnet to deflect the beam to the beam dump, providing the fastest available beam-off response. As a result, a very small amount of MU is delivered in the vicinity of, but not at, the nominal spot position.
- Break Spot – A break spot triggers beam off. Break spots may be either planned in the treatment planning system (TPS) or generated on-the-fly by the control system. A planned break spot is a spot at which the beam is intentionally turned off during the transition from the preceding spot, such that no MU are delivered during transit and the full planned monitor units are delivered at the planned spot position. Planned break spots are used to limit excessive transit dose or to completely avoid transit dose at specific locations. An on-the-fly break spot is a dynamically generated break spot introduced by the control system under predefined delivery conditions to mitigate potential dose deviations and safety risks caused by machine delivery fluctuations.
- Skip Spot – spot for which MU delivery at the planned spot position is skipped because the planned MU is reached prior to the beam arriving at the planned spot position due to delivery fluctuations.
- Spot Position Monitor (SPM) Judgement – verification of spot position (x,y) and spot full width half maximum (FWHM) compared to planned values based on measurement by the SPM. SPM judgement should consider only signals at the planned spot position and exclude signals collected during beam transit.
- Qmon – minimum MU required to provide adequate signal for SPM judgement (Qmon = 0.05 mMU).
- Tmon – minimum time required for SPM judgement.
- Skip SPM judgement – when the stop MU is less than Qmon, the SPM judgment is skipped because the signal is insufficient to reliably assess the beam position and FWHM.
- Cumulative Skipped SPM Judge MU – MU delivered without SPM judgement.

The combination of high dose rate, small spot size, reduced minimum monitor units, and limited time for spot position and FWHM calculation necessitates additional consideration and validation of the beam delivery monitor system. The novel features of the irradiation control system require a thorough prospective risk analysis to ensure efficient and safe system design and performance.

### 2.2 MCF DDCS failure modes and effects analysis

The key steps of the DDCS treatment delivery workflow were first defined and incorporated into a process map. Only the key processes directly related to the DDCS irradiation control system were included, as including all steps necessary for treatment delivery would create an overly complex process map. Many key components of treatment delivery, such as patient positioning and particle acceleration and extraction were not included. The process map began with beam on and concluded with any beam off, whether planned or unplanned.

With the process map defined, each step of the process was then reviewed for potential failure modes by a core group of three medical physicists familiar with the MCF proton delivery system and DDCS delivery process. Due to the complexity and novelty of the MCF system, review of the process map and failure modes was performed collaboratively by a small group to ensure the delivery system details were understood by all involved. Failure mode identification was focused on potential failure modes associated with proton DDCS treatment delivery.

Each potential failure mode was then scored based on the Occurrence (O), Severity (S), and Lack of Detectability (D). Scoring was performed collectively by the core group of physicists and consensus scores were agreed upon. The scoring methodology and scale defined in AAPM Task Group 100 were utilized (19). A modified scoring table was defined to incorporate DDCS specific failure categories such as treatment aborts and on fly break spots (**Table 3**). All failure modes were mapped to one of the scores provided in **Table 3** to ensure consistent and reproducible O, S, and D values for the failure modes investigated. The Risk Priority Number (RPN) of each failure mode was then calculated as the product of the O, S, and D values.

**Table 3:** Scoring table utilized to determine O, S, and D values for RPN calculation.

Rank	Occurrence (O)	Severity (S)	Lack of detectability (D)
2	Failure unlikely, control system fault, delivery system spike	Inconvenience	Very easily detected, automated detection
4	Relatively few failures, calibration error	Minor dosimetric error, on fly break spot	Routine QA performed will detect
6	Occasional failures	Limited toxicity, treatment abort	Detected through manual check, Limited routine QA to detect
8	Repeated failures	Major dosimetric error	Requires good catch to detect, specific QA to detect does not currently exist
10	Failures inevitable	Regulatory concern	Cannot detect

Initial failure mode scoring was performed twice utilizing two separate underlying assumptions. The first scoring was performed assuming no deliberate quality management measures (No QM), such as clinical commissioning and routine machine quality assurance, were incorporated into the process. This assumption is recommended by TG 100 to avoid confusion from the presence of pre-existing quality management strategies. A second scoring was performed with the assumption that clinical commissioning and routine machine quality assurance testing as specified by the American Association of Physicists in Medicine (AAPM) Task Group 185 and AAPM Task Group 224 would be performed (TG QM) (24, 25). Two sets of assumptions were utilized to ensure risks due to both limitations in the overall process and gaps in current guidance documents could be identified. The scoring methodology utilized by the core group of physicists was also reviewed by the entire group of medical physicists at our institution to minimize bias within the smaller scoring group. Note that patient specific quality assurance (PSQA) was assumed to not be incorporated in the QM program for all RPN scoring. The purpose of this analysis is to use the results to inform the development of a comprehensive commissioning plan; therefore, any gaps should be identified and addressed during commissioning rather than relying on PSQA for mitigation.

The failure modes were then sorted by decreasing RPN and further analysis was focused on the failure modes with the greatest RPN values to develop a commissioning program for the incorporation of proton DDCS into clinical practice. Recommendations were focused on two main categories: machine commissioning and treatment planning system (TPS) commissioning.

RPN scoring was then repeated with the assumption that the developed recommendations would be incorporated into the commissioning program for the MCF proton DDCS treatment delivery system.

## 3 Results

### 3.1 MCF DDCS process map

**Figure 2:**
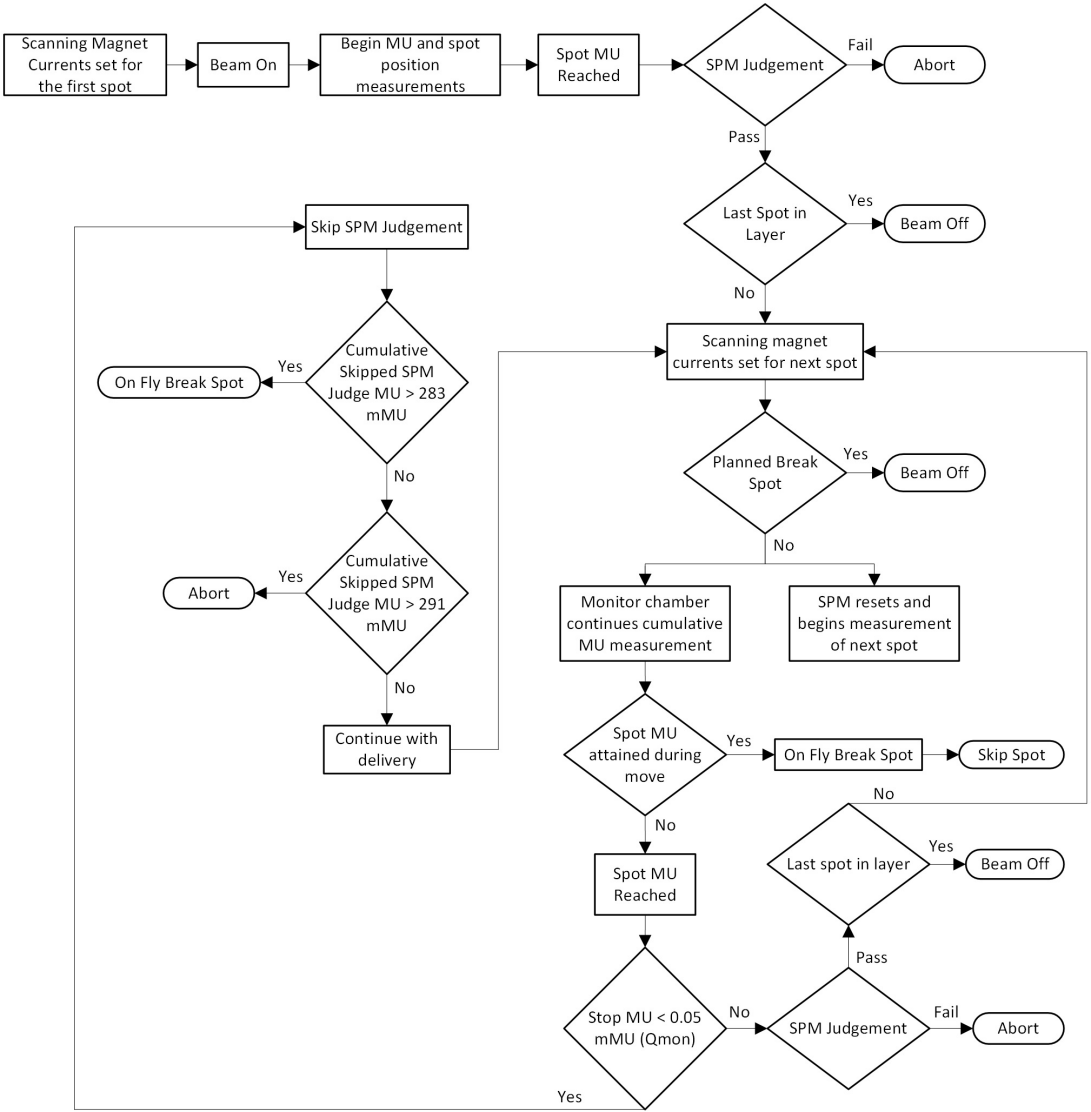
MCF proton DDCS process map. The process map defines the MCF DDCS delivery workflow for a single segment of continuous radiation delivery.

**Figure 2** depicts the MCF Proton DDCS process map for a single segment of radiation delivery until the beam is interrupted by the irradiation control system. The main components of the process map include the MU delivery and SPM judgement processes. The process map includes processes (squares), decisions (diamonds), and ending (oval) shapes to help the reader clearly identify each step in the map.

### 3.2 MCF DDCS failure modes and effects analysis

For each step in the process map, potential failure modes were identified as part of the FMEA. These failure modes arise from different sources within the defined workflow, including failures related to treatment planning system (TPS) modeling limitations, treatment delivery system calibration errors, and delivery instabilities that cause parameters such as scan speed or beam current to fall outside normal variation ranges. Additional failure modes involving control system malfunctions are also included in the analysis. A total of 29 potential failure modes were identified. RPN scoring was performed using the modified scoring table, with RPN values ranging from 24 to 512. The 10 highest RPN scoring failure modes are provided (**Table 4**). A table containing all investigated failure modes can be found in the **Supplementary Material**. Note that the RPN scores for the No QM and TG QM assumptions are identical for multiple failure modes as current task group guidance does not address all DDCS-specific failure modes.

**Table 4:** Failure modes for the 10 highest RPN values for the no QM assumption.

Failure mode	No QM				TG QM			
	O	S	D	RPN	O	S	D	RPN
Scan path and scan time not modeled in TPS (systematic)	8	8	8	512	8	8	8	512
Move dose not modeled in the TPS (systematic)	8	8	8	512	8	8	8	512
Inaccurate MU measurement due to non-negligible dose monitor recombination for high dose rate proton beam (systematic)	6	8	8	384	6	8	6	288
Inaccurate MU measurement due to dose monitor calibration error (systematic)	4	8	8	256	4	8	4	128
Flap dose not modeled in the TPS (systematic)	6	4	8	192	6	4	8	192
Abort due to spot time less than Tmon (the minimum time required for SPM judge)	4	6	8	192	4	6	8	192
Abort due to SPM judgement starting before the beam reaches the planned spot position due to control system scanning time lookup table error	4	6	8	192	4	6	8	192
Abort due to deviation in spot position or spot profile measured in the spot position monitor from the planned value exceeding tolerance	4	6	8	192	4	6	4	96
Inaccurate machine output due to dose monitor drift	4	6	8	192	4	6	4	96
Abort does not occur as expected when cumulative skip SPM judge MU exceed the abort tolerance	2	10	8	160	2	10	8	160

**Figure 3:**
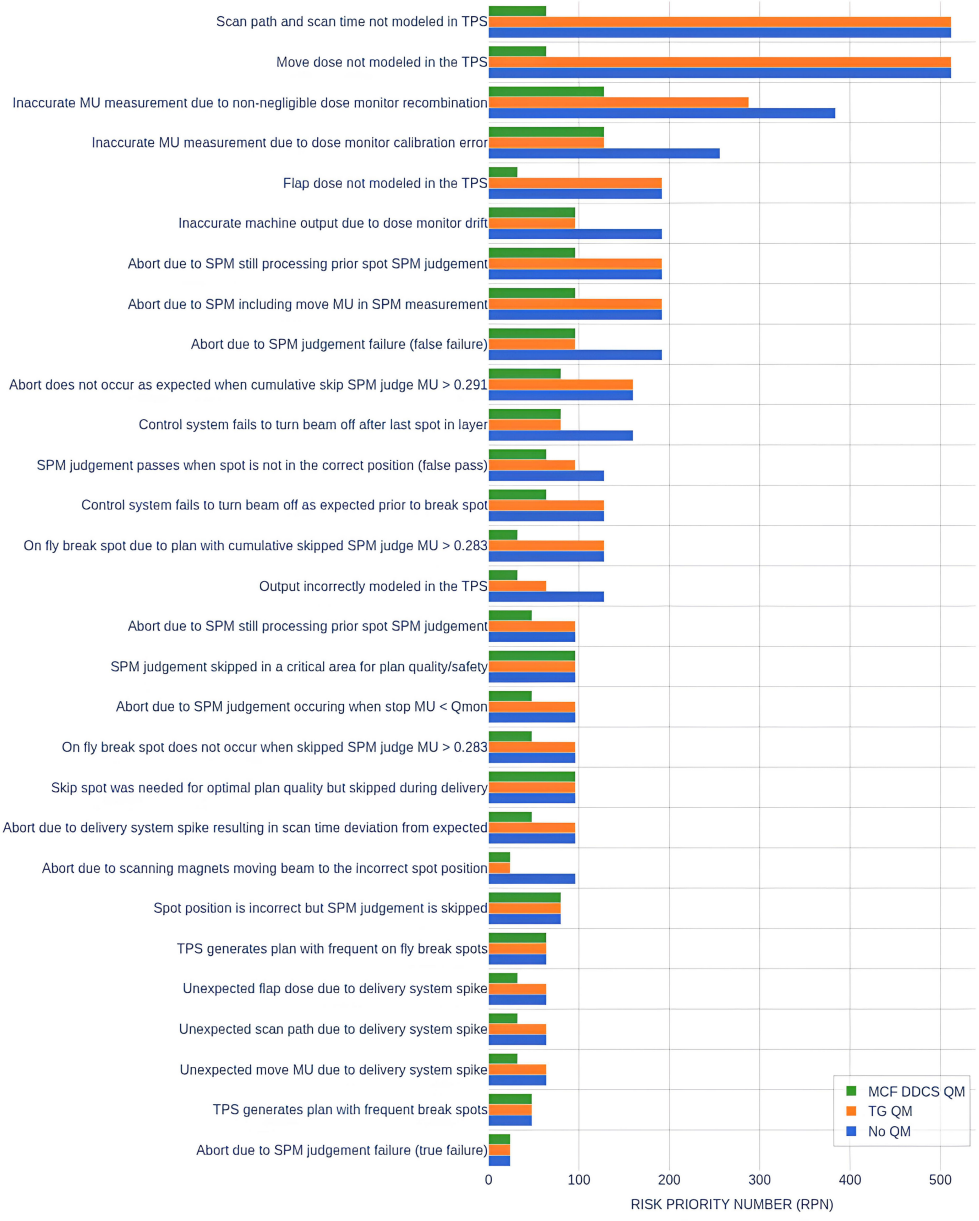
RPN scores for the 29 failure modes identified. RPN scores are presented with assumptions of no quality management (no QM), implementation of task group recommended quality management (TG QM), and implementation of proposed MCF quality management (MCF DDCS QM).

Commissioning plan recommendations were generated to reduce the lack of detectability (D) of the defined failure modes (**Table 5**). Repeat RPN scoring was performed with the assumption that the QM recommendations would be implemented (**Figure 3**). For the 10 highest-scoring failure modes, the proposed QM reduced the mean RPN by 63% and 46% compared to the No QM and TG QM RPN scores, respectively. For all 29 failure modes, the proposed QM reduced the mean RPN by 59% and 52% compared to the No QM and TG QM RPN scores, respectively.

**Table 5:** Summary of proposed additional commissioning items specifically designed for MCF proton DDCS.

Failure mode	Commissioning plan
Scan path and scan time not modeled in TPS (systematic)	Measure scan path and scan time, using task-specific spot map. Develop analytical model for scan path and scan time. Implement the model in the TPS. Measure scan path and scan time, with spot maps and energies covering the clinical range, and compare it with TPS prediction.
Move dose not modeled in the TPS (systematic)	Implement model for move dose in the TPS based on scan time and scan path model. Specifically, the move MU is defined as the product of the scan time and the planned beam current (MU/s), with its deposition distributed along the scan path. Measure move dose, with spot maps and energies covering the clinical range. Compare TPS calculations with measurements to verify move dose is accurately modeled in the TPS.
Inaccurate MU measurement due to non-negligible ion recombination in the dose monitor for high dose rate proton beam (systematic)	Measure dose monitor collection efficiency for the clinical range of energy and beam current, focusing on high beam current and high energy proton beams. Verify proper corrections are applied by the delivery system to keep dose monitor measurement accurate within the clinical range energies and beam currents.
Inaccurate MU measurement due to dose monitor calibration error (systematic)	Follow standard recommended protocol; Follow TRS-398 for reference dose calibration (29). Use IROC for independent output audit and phantom accreditation.
Flap dose not modeled in the TPS (systematic)	Characterize flap time (the time from when beam-off is executed to the time that dose monitor does not receive any counts) using machine log files. Model the flap MU (flap time * beam current) in TPS.
Abort due to spot time less than Tmon (the minimum time required for SPM judge)	In the TPS, include spot time checking function to remove spots with a spot time less than Tmon and recalculate the final dose.
Abort due to SPM judgement starting before the beam reaches the planned spot position due to control system scanning time lookup table error	Measure scan time, with spots maps and energies covering the clinical range and including edge cases. Ensure the control system scan time model/lookup table is accurate.
Abort due to deviation in spot position or spot profile measured in the spot position monitor from the planned value exceeding tolerance	Follow standard recommended protocol; Follow TG 185 to model the spatial and angular distribution of the beam (24). Follow TG 224 to perform daily, monthly and annual QA for spot position and spot size (25).
Inaccurate machine output due to dose monitor drift	Follow standard recommended protocol; Specially, A daily QA output deviation greater than 2% from baseline will trigger an investigation; if the deviation is confirmed to be due to dose monitor drift, an output adjustment will be performed.
Abort does not occur as expected when cumulative skip SPM judge MU exceed the abort tolerance	Develop a specific test plan with a move MU exceeding 0.291 MU. As there is no SPM judgment for move MU, this plan is intended to verify that the irradiation control system operates as expected.

## 4 Discussion

This work outlines the MCF proton DDCS delivery irradiation control system and the prospective risk analysis performed prior to the commissioning and clinical implementation of the system. It provides the first detailed description of the MCF proton system, which is designed exclusively for DDCS treatment delivery with a unique irradiation control system design. The methodology of TG 100 was followed where possible, but the task group report is only able to provide general guidance and cannot inform the reader of the specifics for their individual system. A modified RPN scoring table was generated to allow for consistent scoring within the scoring group and systematically match failures to an assigned score. Because this prospective FMEA analysis is on a first-of-its kind system, determining O, S, and D values proved challenging for a system which has not been previously implemented clinically, as the scoring team was unable to rely on experience with the system to determine appropriate scoring values. Identification of failure modes also proved challenging as prior failures of the system do not yet exist. The scoring team relied on their clinical judgement, experience with analogous systems, and detailed understanding of the MCF control system design to estimate potential failures. While FMEA is often applied for existing processes to better catch failure modes, this work was conducted in a truly prospective manner to identify and mitigate failures before clinical use.

The failure modes with the largest initial RPN scores were primarily related to delivery features unique to DDCS, namely the move dose and the location of the move dose deposition during transit. In other particle therapy systems that implement DDCS delivery, the move dose is often not accounted for in the TPS and must be limited to minimize the dose contribution that is not considered in the calculation. Common mitigation strategies include reducing the beam current and introducing breakpoint strategies to limit the contribution of move dose (26). Reducing the beam current decreases the move MU, since the move dose is the product of beam current and scan time. As a result, for reduced beam currents the majority of the planned spot MU is delivered at the stationary spot position, and hence the dose deviation caused by neglecting the move dose within the TPS remains within clinically acceptable tolerances (4). While these approaches effectively mitigate move dose related uncertainties, they do so at the cost of slower treatment delivery and reduced delivery efficiency. In contrast, the MCF particle therapy system adopts a different strategy: rather than slowing treatment delivery, we aim to fully commission and explicitly account for the move dose within the delivery and dose calculation framework. The magnitude and location of the move dose depend on the scan path and time, therefore, the scan path and scan time need to be measured and modeled during commissioning. The performance of the delivery system and agreement with the TPS must be thoroughly validated during initial commissioning and routinely checked during routine machine quality assurance. Once the scan time is modeled, the move MU can be calculated as the product of scan time and beam current. The calculated move MU will then to be validated through measurement, which requires a detector with high spatial and temporal resolution to measure the beam during the move. Tsubouchi et al. and Chua et al. utilized a 2D detector array for DDCS measurements of carbon ion and proton beams (10, 11, 27). The inclusion of similar measurements during commissioning significantly dropped the MCF DDCS QM RPN values as these items would be incorporated into the MCF commissioning plan.

Multiple failure modes related to the beam output were also evaluated. TG QM effectively verifies the accuracy of the dose monitor under routine conditions, but the MCF system will incorporate a wide range of dose rates and spot sizes beyond those typically seen in other proton therapy systems, which can affect the collection efficiency of the dose monitor chamber. The delivery system will incorporate internal corrections to account for recombination due to dose rate and spot size, but the corrections must be thoroughly validated during initial commissioning and verified through routine quality assurance.

In DSS, the SPM measures all delivered MU as all MU are delivered to the planned spot positions. With DDCS the SPM only measures the stop MU to avoid including MU delivered while the beam is moving. This requires the SPM to precisely know, based on the scan time model, which portion of treatment delivery to include in the measurements used for SPM judgement. Multiple failure modes related to SPM judgement and SPM measurement were identified that would result in a treatment abort due to a failure of the SPM judgement. Validation of the SPM for both stationary and moving spots during commissioning is required to ensure that the SPM can accurately perform a judgement under all clinical scenarios.

The MCF control system incorporates additional safety features specially designed to initiate on fly break spots or treatment aborts under certain conditions as previously discussed. The ability of the system to interrupt treatment delivery as designed must be verified during initial commissioning and with routine safety checks. Test plans can be designed to generate the conditions under which delivery system intervention is needed but recreating all scenarios in which the delivery system will intervene may not be possible as unanticipated delivery characteristics, such as large beam current fluctuations, cannot be easily reproduced within the delivery system.

It is worth noting that there may be additional failure modes that were not foreseen during the prospective risk assessment performed. The MCF proton DDCS system incorporates unique features related to both treatment delivery and treatment planning for which there is limited clinical experience that can be relied upon. RPN scoring was performed without prior operational experience with the system, and the FMEA analysis performed focuses on only a small portion of the overall treatment delivery workflow. Other systems may have their own unique features that result in potential failure modes not applicable to our system. The designed commissioning plan may need adjustment once the system is operational, and the developed quality management program must be routinely reviewed after clinical implementation to ensure that the QM program effectively mitigates risk and ensures patients are treated safely and effectively.

## 5 Conclusions

This work presents a comprehensive description and prospective risk analysis of the Mayo Clinic Florida proton dose−driven continuous scanning irradiation control system, performed prior to clinical commissioning of this novel delivery platform. The findings underscore the importance of rigorous, system−specific evaluation for emerging delivery techniques that fall outside the scope of existing guidance documents. As clinical experience with proton DDCS grows, the commissioning framework and quality−management recommendations established here will support safe and efficient treatment delivery. Continued review of system behavior during commissioning and after clinical deployment will be essential to ensure that the commissioning plan and quality−management program remains responsive to unforeseen potential failures, real−world delivery characteristics, and evolving clinical practice.

## Data Availability

The original contributions presented in the study are included in the article/**Supplementary Material**. Further inquiries can be directed to the corresponding author.
