# Supplementary material for: A prospective risk analysis for the clinical commissioning of a dose-driven continuous scanning proton therapy system
Source: Front Oncol. 2026 May 20;16:1826522. doi: 10.3389/fonc.2026.1826522 (PMC13229732; doi:10.3389/fonc.2026.1826522)
Supplement: Supplementary file 1 [file Table1.docx]

Supplemental Table: Failure mode scores for the 29 investigated failure modes. Failure modes were scored assuming no quality management (No QM), task group recommended quality management (TG QM), and with the proposed commissioning items identified in this work (MCF DDCS QM).

|  | **No QM** | | | | **TG QM** | | | | **MCF DDCS QM** | | | |
| --- | --- | --- | --- | --- | --- | --- | --- | --- | --- | --- | --- | --- |
| **Failure Mode** | **O** | **S** | **D** | **RPN** | **O** | **S** | **D** | **RPN** | **O** | **S** | **D** | **RPN** |
| Scan path and scan time not modeled in TPS | 8 | 8 | 8 | 512 | 8 | 8 | 8 | 512 | 2 | 8 | 4 | 64 |
| Move dose not modeled in the TPS | 8 | 8 | 8 | 512 | 8 | 8 | 8 | 512 | 2 | 8 | 4 | 64 |
| Inaccurate MU measurement due to non-negligible dose monitor recombination | 6 | 8 | 8 | 384 | 6 | 8 | 6 | 288 | 4 | 8 | 4 | 128 |
| Inaccurate MU measurement due to dose monitor calibration error | 4 | 8 | 8 | 256 | 4 | 8 | 4 | 128 | 4 | 8 | 4 | 128 |
| Flap dose not modeled in the TPS | 6 | 4 | 8 | 192 | 6 | 4 | 8 | 192 | 2 | 4 | 4 | 32 |
| Inaccurate machine doutput due to dose monitor drift | 4 | 6 | 8 | 192 | 4 | 6 | 4 | 96 | 4 | 6 | 4 | 96 |
| Abort due to SPM still processing prior spot SPM judgement | 4 | 6 | 8 | 192 | 4 | 6 | 8 | 192 | 2 | 6 | 8 | 96 |
| Abort due to SPM including move MU in SPM measurement | 4 | 6 | 8 | 192 | 4 | 6 | 8 | 192 | 4 | 6 | 4 | 96 |
| Abort due to SPM judgement failure (false failure) | 4 | 6 | 8 | 192 | 4 | 6 | 4 | 96 | 4 | 6 | 4 | 96 |
| Abort does not occur as expected when cumulative skip SPM judge MU > 0.291 | 2 | 10 | 8 | 160 | 2 | 10 | 8 | 160 | 2 | 10 | 4 | 80 |
| Control system fails to turn beam off after last spot in layer | 2 | 10 | 8 | 160 | 2 | 10 | 4 | 80 | 2 | 10 | 4 | 80 |
| SPM judgement passes when spot is not in the correct position (false pass) | 4 | 4 | 8 | 128 | 4 | 4 | 6 | 96 | 4 | 4 | 4 | 64 |
| Control system fails to turn beam off as expected prior to break spot | 2 | 8 | 8 | 128 | 2 | 8 | 8 | 128 | 2 | 8 | 4 | 64 |
| On fly break spot due to plan with cumulative skipped SPM judge MU > 0.283 | 4 | 4 | 8 | 128 | 4 | 4 | 8 | 128 | 4 | 4 | 2 | 32 |
| Output incorrectly modeled in the TPS | 2 | 8 | 8 | 128 | 2 | 8 | 4 | 64 | 2 | 8 | 2 | 32 |
| Abort due to SPM still processing prior spot SPM judgement | 2 | 6 | 8 | 96 | 2 | 6 | 8 | 96 | 2 | 6 | 4 | 48 |
| SPM judgement skipped in a critical area for plan quality/safety | 4 | 4 | 6 | 96 | 4 | 4 | 6 | 96 | 4 | 4 | 6 | 96 |
| Abort due to SPM judgement still happening when stop MU < Qmon | 2 | 6 | 8 | 96 | 2 | 6 | 8 | 96 | 2 | 6 | 4 | 48 |
| On fly break spot does not occur when cumulative skipped SPM judge MU > 0.283 | 2 | 6 | 8 | 96 | 2 | 6 | 8 | 96 | 2 | 6 | 4 | 48 |
| Skip spot was needed for optimal plan quality but skipped during delivery | 4 | 4 | 6 | 96 | 4 | 4 | 6 | 96 | 4 | 4 | 6 | 96 |
| Abort due to delivery system spike resulting in scan time deviation from expected | 2 | 6 | 8 | 96 | 2 | 6 | 8 | 96 | 2 | 6 | 4 | 48 |
| Abort due to scanning magnets moving beam to the incorrect spot position | 2 | 6 | 8 | 96 | 2 | 6 | 2 | 24 | 2 | 6 | 2 | 24 |
| Spot position is incorrect but SPM judgement is skipped | 2 | 4 | 10 | 80 | 2 | 4 | 10 | 80 | 2 | 4 | 10 | 80 |
| TPS generates plan that results in frequent on fly break spots | 4 | 2 | 8 | 64 | 4 | 2 | 8 | 64 | 4 | 2 | 8 | 64 |
| Unexpected flap dose delivery due to delivery system spike | 2 | 4 | 8 | 64 | 2 | 4 | 8 | 64 | 2 | 4 | 4 | 32 |
| Unexpected scan path due to delivery system spike | 2 | 4 | 8 | 64 | 2 | 4 | 8 | 64 | 2 | 4 | 4 | 32 |
| Unexpected move MU delivered due to delivery system spike | 2 | 4 | 8 | 64 | 2 | 4 | 8 | 64 | 2 | 4 | 4 | 32 |
| TPS generates plan that results in frequent break spots | 4 | 2 | 6 | 48 | 4 | 2 | 6 | 48 | 4 | 2 | 6 | 48 |
| Abort due to SPM judgement failure (true failure) | 2 | 6 | 2 | 24 | 2 | 6 | 2 | 24 | 2 | 6 | 2 | 24 |
